# Cortical thinning in relation to impaired insight into illness in patients with treatment resistant schizophrenia

**DOI:** 10.1038/s41537-023-00347-y

**Published:** 2023-04-29

**Authors:** Julia Kim, Jianmeng Song, Yasaman Kambari, Eric Plitman, Parita Shah, Yusuke Iwata, Fernando Caravaggio, Eric E. Brown, Shinichiro Nakajima, M. Mallar Chakravarty, Vincenzo De Luca, Gary Remington, Ariel Graff-Guerrero, Philip Gerretsen

**Affiliations:** 1 Multimodal Imaging Group, Research Imaging Centre, Centre for Addiction and Mental Health (CAMH), Toronto, ON Canada; 2 Institute of Medical Science, University of Toronto, Toronto, ON Canada; 3 Cerebral Imaging Centre, Douglas Mental Health University Institute, McGill University, Montreal, QC Canada; 4 Department of Psychiatry, McGill University, Montreal, QC Canada; 5 University of Yamanashi, Faculty of Medicine, Department of Neuropsychiatry, Yamanashi, Japan; 6 Department of Psychiatry, University of Toronto, Toronto, ON Canada; 7 Campbell Family Mental Health Research Institute, CAMH, Toronto, ON Canada; 8 Geriatric Mental Health Division, CAMH, Toronto, ON Canada; 9 Department of Neuropsychiatry, Keio University School of Medicine, Tokyo, Japan; 10 Department of Biomedical Engineering, McGill University, Montreal, QC Canada; 11 Schizophrenia Division, CAMH, Toronto, ON Canada

**Keywords:** Biomarkers, Schizophrenia

## Abstract

Impaired insight into illness is a common element of schizophrenia that contributes to treatment nonadherence and negative clinical outcomes. Previous studies suggest that impaired insight may arise from brain abnormalities. However, interpretations of these findings are limited due to small sample sizes and inclusion of patients with a narrow range of illness severity and insight deficits. In a large sample of patients with schizophrenia, the majority of which were designated as treatment-resistant, we investigated the associations between impaired insight and cortical thickness and subcortical volumes. A total of 94 adult participants with a schizophrenia spectrum disorder were included. Fifty-six patients (60%) had treatment-resistant schizophrenia. The core domains of insight were assessed with the VAGUS insight into psychosis scale. We obtained 3T MRI T1-weighted images, which were analysed using CIVET and MAGeT-Brain. Whole-brain vertex-wise analyses revealed impaired insight, as measured by VAGUS average scores, was related to cortical thinning in left frontotemporoparietal regions. The same analysis in treatment-resistant patients showed thinning in the same regions, even after controlling for age, sex, illness severity, and chlorpromazine antipsychotic dose equivalents. No association was found in non-treatment-resistant patients. Region-of-interest analyses revealed impaired general illness awareness was associated with cortical thinning in the left supramarginal gyrus when controlling for covariates. Reduced right and left thalamic volumes were associated with VAGUS symptom attribution and awareness of negative consequences subscale scores, respectively, but not after correction for multiple testing. Our results suggest impaired insight into illness is related to cortical thinning in left frontotemporoparietal regions in patients with schizophrenia, particularly those with treatment resistance where insight deficits may be more chronic.

## Introduction

Moderate-to-severe impairment of insight into illness commonly occurs in schizophrenia and contributes to treatment nonadherence and negative clinical outcomes^1,2^. Impaired insight is frequently associated with poor adherence to pharmacological treatment^3–6^, worse symptom severity^7–9^, and increased rates of relapse and hospitalisation^10,11^. Despite its clinical relevance, the pathophysiological mechanism(s) of impaired insight in patients with schizophrenia remain elusive.

Previous structural neuroimaging studies suggest that impaired insight may arise from underlying brain abnormalities. Impaired insight has been commonly associated with reduced brain volume in frontal regions^12,13^, but also temporal^14^, parietal, occipital^15^, and subcortical brain areas^16^. A recent coordinate-based meta-analysis similarly reported abnormalities in isolated brain-regions in relation to impaired insight, but with spatially diffuse global and frontal abnormalities^17^. However, the results of these earlier studies are largely inconsistent with some studies finding no structural correlates of impaired insight^14,18,19^.

Some explanations for the inconsistencies in the literature include small sample size, heterogeneity in patient samples, and inclusion of patients with a narrow range of illness severity and insight deficits. Studies have also used various measures of insight, with some using single- versus multi-item scales to assess only a single or multiple dimensions of insight into illness^17,19^, making it difficult to integrate and interpret these findings.

This study attempted to address some of the limitations of earlier studies. The aims were to assess the associations between impaired insight into illness and (i) cortical thickness, and (ii) regional subcortical volumes in patients with schizophrenia, the majority of which had treatment resistance. Treatment-resistant patients, as defined by partial or no response to first-line antipsychotic medications, i.e., typical or atypical non-clozapine antipsychotic drugs, are reported to have widespread reductions in cortical and subcortical volumes and cortical thickness^20–22^. We are not aware of any study that has explored the association between impaired insight and cortical thickness in this population. We employed the VAGUS scale to assess insight to illness, which is a brief, yet comprehensive measure that assesses the four core dimensions of insight, including general illness awareness, symptom attribution, awareness of the need for treatment, and awareness of the negative consequences of the disorder^23^.

We hypothesised cortical thinning in frontoparietal regions in relation to impaired insight with more pronounced thinning in these regions in patients with treatment-resistant schizophrenia. We also hypothesised a relationship between impaired insight and reduced subcortical volumes, as deficits in subcortical regions are widely reported in patients with schizophrenia^19^.

## Methods

### Study participants

In this cross-sectional study, inpatients or outpatients ≧18 years of age with a DSM-IV/Structured Clinical Interview (SCID-4)^24^ diagnosis of schizophrenia or schizoaffective disorder were recruited at the Centre for Addiction and Mental Health (CAMH), Toronto, Canada from 2014 to 2019. The data for this analysis was acquired from two studies: (1) a cross-sectional study investigating glutamatergic neurometabolite levels in treatment-resistant schizophrenia^25^, and (2) an intervention study that examined the effects of transcranial direct current stimulation on impaired insight in patients with schizophrenia (ClinicalTrials.gov Identifier: NCT02848885). The SCID was administered by two clinicians (YI and SN). Treatment resistance was defined as a suboptimal response to at least 2 previous non-clozapine antipsychotics, each attaining a chlorpromazine (CPZ) equivalent dose of 400 mg or more for 6 or more consecutive weeks based on the modified Treatment Response and Resistance in Psychosis Working Group Consensus criteria^26–28^. Both studies were approved by the CAMH Research Ethics Board. Capacity to consent to participate in the study was confirmed with the MacArthur Test of Competence^29^.

Exclusion criteria for both studies included: being unable to provide consent to participate in the research study; substance abuse or dependence within one month prior to entering the study (except caffeine and nicotine); a positive urine drug test for illicit drugs; any contraindication to magnetic resonance imaging (MRI); serious unstable medical illness or any concomitant major medical or neurological illness, including a history of seizures or a first degree relative with a history of a seizure disorder.

### Study measures

Insight was measured using the VAGUS insight into psychosis scale (www.vagusonline.com), self-report version^23^. The VAGUS scale has four subscales that assess the core components of insight, including general illness awareness, accurate symptom attribution, awareness of the need for treatment, and awareness of negative consequences of the disorder. Each subscale score ranges from 0 to 10, with a higher score representing greater insight. An average score is derived from the subscale scores. The VAGUS self-report measure is stongly associated with the VAGUS clinician-rated version (*r* = 0.70 and *p* < 0.001)^23^. Illness severity was assessed using the Positive and Negative Syndrome Scale (PANSS). The PANSS total score was modified to exclude the PANSS G12 lack of judgement and insight item^30^. The WRAT-III reading subtest was used to measure premorbid IQ^31^. The R package, chlorpromazineR (version 0.1.2), was used to determine CPZ antipsychotic drug dose equivalents^32^. Self-reported smoking status was also collected.

### Statistical analysis for demographic and clinical characteristics

SPSS Statistics version 21 (IBM Corporation) was used to carry out descriptive analyses for demographic and clinical data. Independent *t*-tests, *χ*^2^, and Fisher’s exact tests were employed to compare the demographic and clinical characteristics of treatment-resistant and non-treatment-resistant patients where appropriate. The significance level for these tests was set at *p* < 0.05.

### Magnetic resonance imaging acquisition and processing

Three-dimensional IR-prepared T1-weighted MRI scans (BRAVO, TE = 3.00 ms, TR = 6.74 ms, TI = 650 ms, flip angle = 8°, FOV = 23 cm, 256 × 256 matrix, slice thickness = 0.9 mm) were obtained at CAMH using a 3T GE Discovery MR750 scanner (General Electric, Waukesha, WI).

T1-weighted images were preprocessed using the bpipe pipeline (http://github.com/CobraLab/minc-bpipe-library/), which consists of bias field correction^33^, neck cropping, and Brain Extraction based on nonlocal Segmentation Technique (BEaST) brain extraction^34^. All preprocessed images were visually inspected for quality control.

Cortical thickness analysis was performed using the CIVET processing pipeline (version 2.1.0; Montreal Neurological Institute). This procedure has been previously described in detail elsewhere^35–37^. Briefly, preprocessed T1-weighted images were linearly registered to the ICBM 152 average template using a nine-parameter transformation (i.e., 3 translations, rotations, and scales)^38^, and classified into grey matter (GM), white matter (WM) and cerebrospinal fluid^39,40^. The GM and WM surfaces were modelled onto the left and right hemispheres, each composed of 163,842 vertices^41,42^. Cortical thickness was determined by evaluating the distance between the WM and GM surfaces in the native space^43,44^. A 30 mm surfaced based diffusion kernel for vertex-wise analysis was used for smoothing and the results were nonlinearly registered to a surface template^45^. Regions of interest (ROI) were generated based on the Automated Anatomical Labelling (AAL) atlas^46,47^. ROI data was extracted from unsmoothed data. CIVET outputs were visually inspected for quality control.

Multiple Automatically Generated Templates (MAGeT-Brain) algorithm was used to carry out a fully-automated segmentation of subcortical volumes^48,49^. MAGeT-Brain is described in detail elsewhere^19,50–53^. The Colin-27 Subcortical Atlas^54,55^ was used in this analysis to obtain volumes of the striatum, thalamus, and globus pallidus. From the overall sample, scans from a subset of participants were selected as a template library via which the final segmentation was bootstrapped. In this analysis, 21 templates were used. The bootstrapping of the final segmentations through the template library resulted in the production of candidate labels for each subject. Candidate labels were then fused using a majority vote to complete the segmentation process. A version of the Automatic Normalization Tools (ANTS) registration technique, which is compatible with the minc toolkit (https://github.com/vfonov/mincANTS), was used for nonlinear registration^56^.

### Cortical thickness analyses

#### Vertex-wise analyses

Cortical thickness analyses were conducted using the RMINC package (https://github.com/mcvaneede/RMINC). First, whole-brain vertex-wise analyses were carried out using a general linear model for VAGUS average and subscale scores, controlling for age and sex. Maps of t-statistics at each vertex were projected onto an average brain template. Second, for any significant associations, we repeated the whole-brain vertex-wise analysis additionally controlling for illness severity and CPZ antipsychotic dose equivalents. Third, we performed separate analyses for treatment-resistant and non-treatment-resistant participants, including the same covariates. Total brain volume was not controlled for as cortical thickness and brain volume are poorly correlated^57^. Whole-brain analyses were corrected for multiple testing using a false discovery rate (FDR) < 0.10^58^. A lower threshold was used to reveal all affected brain regions.

#### Regions of interest analyses

ROI analyses were conducted using SPSS. The mean cortical thickness was generated for our ROIs based on the AAL atlas^46,47^. The supramarginal gyrus and the angular gyrus were selected as the a priori ROIs based on findings from previous neuroimaging studies that examined insight into illness in patients with schizophrenia^59–61^. Regression analyses were carried out to assess the relationship between VAGUS average and subscale scores with cortical thickness in the supramarginal and angular gyrus as separate dependent variables. The same covariates used in the vertex-wise analyses were included in the ROI analyses, i.e., age, sex, illness severity, and CPZ antipsychotic dose equivalents. The analyses were repeated for treatment-resistant and non-treatment-resistant patients. The threshold of significance level was established at *p* < 0.006 (0.05/8 (i.e., 2 separate regression models for each of the 4 ROIs)).

### Subcortical volume analyses

Regression analyses were carried out using SPSS to assess the relationship between VAGUS average and subscale scores with left and right striatal, thalamic, and globus pallidus volumes^62,63^. Age, sex, illness severity, TBV, and CPZ antipsychotic dose equivalents were included as covariates. The analyses were repeated for treatment-resistant and non-treatment-resistant patients. The threshold of significance level was established at *p* < 0.004 (0.05/12 (i.e., 2 separate regression models for each of the 6 subcortical ROIs)).

## Results

### Demographic and clinical characteristics

A total of 94 participants were included. The demographic and clinical characteristics can be found in Table 1. Correlations between VAGUS scores and other demographic and clinical characteristics are shown in Supplemental Material 1. Supplemental Material 2 lists the antipsychotic medications used by participants.

**Table 1:** Participant (*n* = 94) demographic and clinical characteristics.

	Mean (SD) or *N*, Range			
	All patients (*N* = 94)	Treatment-Resistant (*N* = 56)	Non-Treatment Resistant (*N* = 38)	t(df) or *χ*^2^, *p*-value^a^
Age (years)	42.5 (12.3), 19.0-69.0	42.6 (12.0), 23.0-69.0	42.4 (13.0), 19.0-66.0	t(92) = -0.058, *p* = 0.954
Sex (male/female)	67/27	43/13	24/14	*χ*^2^(94) = 2.05, *p* = 0.152
Diagnosis (SCZ/SA)	60/34	36/20	24/14	*χ*^2^(94) = 0.012, *p* = 0.911
Age of onset (years)	23.7 (7.0), 9.0-46.0	22.5 (6.5), 12.0-42.0	23.3 (8.0), 9.0-46.0	t(92) = 0.48, *p* = 0.627
Illness duration (years)	19.6, 2.0-50.0	19.4 (11.6), 2.0-49.0	19.8 (12.4), 3.0-50.0	t(92) = 0.14, *p* = 0.887
Education (years)	12.7 (2.5), 6.0-20.0	12.5 (2.6), 6.0-20.0	13.2 (2.2), 8.0-17.0	t(92) = 1.36, *p* = 0.177
WTAR	100.8 (14.2), 5.0-49.0	99.8 (15.4), 57.0-123.0	102.3 (12.4), 69.0-125.0	t(86) = 0.83, *p* = 0.409
Tobacco use (use/no use)	50/44	28/28	22/16	*χ*^2^(94) = 0.567, *p* = 0.452
VAGUS				
Average	7.1 (1.7), 2.5-10.0	6.9 (1.8), 2.5-10.0	7.3 (1.3), 4.1-9.9	t(92) = 1.11, *p* = 0.270
General illness awareness	7.0 (2.6), 0-10.0	6.9 (2.6), 0-10.0	7.1 (2.5), 0-10.0	t(92) = 0.45, *p* = 0.654
Symptom attribution	5.6 (2.8), 0-10.0	5.7 (2.6), 0-10.0	5.4 (3.1), 0-10.0	t(88) = -0.43, *p* = 0.669
Need for treatment	8.0 (2.1), 0-10.0	7.8 (2.3), 0-10.0	8.2 (1.8), 2.3-10.0	t(91) = 0.86, *p* = 0.393
Negative consequences	7.8 (2.5), 0-10.0	7.4 (2.5), 0-10.0	8.5 (2.5), 1-10.0	t(92) = 2.13, *p* = 0.036
PANSS				
Total modified^b^	63.7 (14.6), 34.0-96.0	65.2 (16.0), 34.0-96.0	61.6 (11.9), 40.0-91.0	t(92) = -1.17, *p* = 0.244
Positive	16.2 (5.9), 7.0-31.0	17.4 (6.2), 7.0-31.0	14.4 (5.0), 8.0-27.0	t(92) = -2.53, *p* = 0.013
Negative	17.5 (4.5), 8.0-29.0	18.2 (5.0), 8.0-29.0	16.6 (3.3), 10.0-23.0	t(92) = -1.74, *p* = 0.085
General	34.1(7.6), 19.0-53.0	34.1 (8.4), 19.0-53.0	34.1 (6.4), 23.0-49.0	t(92) = -0.012, *p* = 0.991
CPZ dose (mg/day)	475.7 (222.0), 37.5-1000.0	505.9 (234.8), 37.5-975.0	439.2 (228.6), 75.0-1000.0	t(92) = -1.37, *p* = 0.175

*SCZ* schizophrenia, *SA* schizoaffective, *CPZ* chlorpromazine, *PANSS* Positive and Negative Syndrome Scale, *WTAR* Wechsler Test of Adult Reading.

^a^Independent *t*-test or chi-squared tests between treatment-resistant and non-treatment-resistant patients.

^b^PANSS total score minus item G12.

### Impaired insight and cortical thickness

#### Whole brain

**Fig. 1:**
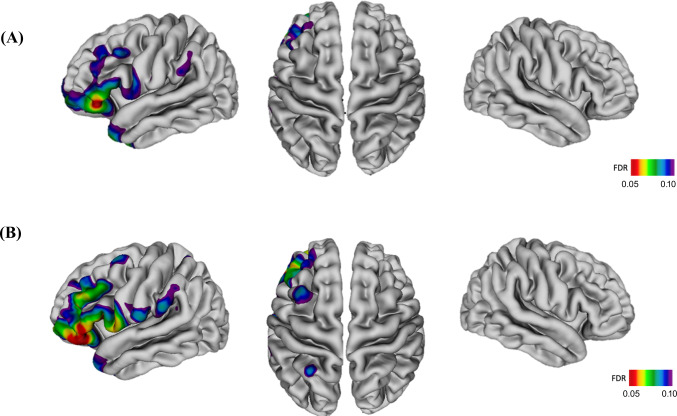
Whole-brain analyses results. **A** Association between cortical thinning and impaired insight as measured by VAGUS average scores in patients with schizophrenia, controlling for age and sex (*n* = 94); **B** Association between cortical thinning and impaired insight as measured by VAGUS average scores in treatment-resistant patients with schizophrenia, controlling for age, sex, illness severity, and chlorpromazine dose equivalents (*n* = 56).

Impaired insight (i.e., lower VAGUS average scores) was associated with cortical thinning in the left middle and inferior frontal regions, temporal pole, and supramarginal gyrus, controlling for age and sex (Fig. 1A). No association was found when additionally controlling for illness severity and CPZ antipsychotic dose. No associations were found between VAGUS subscale scores and cortical thickness.

Subgroup analysis of only treatment-resistant patients showed cortical thinning in the same regions in relation to impaired insight (i.e., lower VAGUS average scores), controlling for age, sex, illness severity, and CPZ antipsychotic drug dose equivalents (Fig. 1B). No associations were found between VAGUS subscale scores and cortical thickness in treatment-resistant patients. There were no associations between VAGUS average and subscale scores and cortical thickness in non-treatment-resistant patients.

#### Regions of interest

There was an association between VAGUS average scores and cortical thickness in the left supramarginal gyrus, but not after correction for multiple testing (standardised *β* = 0.20, *t* = 2.06, *p* = 0.042). Impaired general illness awareness (i.e., lower VAGUS general illness awareness domain score) was associated with cortical thinning in the left supramarginal gyrus (standardised *β* = 0.34, *t* = 2.86, *p* = 0.005) (Table 2). There were no associations between VAGUS average or subscale scores with the right supramarginal gyrus, and the left and right angular gyrus.

**Table 2:** Stepwise linear regression analysis examining cortical thickness in the left supramarginal gyrus with VAGUS subscale scores, age, sex, illness severity, and CPZ dose equivalents as predictors.

				Correlations		
Predictors	Beta	*t*	*p*-value	Zero-order	Partial	Part
Age	–0.51	–5.12	<0.001^*^	–0.44	–0.45	–0.48
Sex	0.00	0.04	0.972	–0.05	0.00	0.00
CPZ equivalent dose	–0.09	–0.92	0.361	–0.08	–0.10	–0.09
PANSS total modified^a^	0.05	0.43	0.667	0.02	0.05	0.04
VAGUS subscales						
General illness awareness	0.34	2.86	0.005*	0.18	0.30	0.27
Symptom attribution	–0.04	–0.38	0.702	0.03	–0.04	–0.04
Need for treatment	–0.10	–0.84	0.406	–0.05	–0.09	–0.08
Negative consequences	0.04	0.43	0.667	0.03	0.05	0.04

*CPZ* chlorpromazine, *PANSS* Positive and Negative Syndrome Scale.

^a^PANSS total score minus item G12.

*p < 0.006

Subgroup analysis of only treatment-resistant patients similarly found an association between lower VAGUS average scores and cortical thinning in the left supramarginal gyrus, but not after correction for multiple testing (standardised *β* = 0.20, *t* = 2.06, *p* = 0.021). There was no relationship between VAGUS subscale scores and cortical thinning in the left supramarginal gyrus in treatment-resistant patients. No associations were found between VAGUS average or subscale scores and the left supramarginal gyrus in non-treatment-resistant patients. In both groups, no relationships were found between VAGUS average or subscale scores and the right supramarginal gyrus, and the left and right angular gyrus.

### Impaired insight and subcortical volume

There was an association between VAGUS symptom attribution subscale scores and right thalamic volume (*t* = –2.14, *p* = 0.035) and VAGUS awareness of negative consequences subscale scores and left thalamic volume (*t* = –2.11, *p* = 0.038), but not after correction for multiple testing. There were no associations between VAGUS average or subscale scores and the striatum, and globus pallidus, bilaterally. The results remained the same when analyzing treatment-resistant and non-treatment-resistant patients separately.

## Discussion

This study examined the relationships between impaired insight and (i) cortical thickness, and (ii) subcortical volumes in patients with a schizophrenia spectrum disorder. We found cortical thinning in the left inferior and middle frontal regions, supramarginal gyrus, and the temporal pole in relation to impaired insight, particularly in treatment-resistant patients where insight deficits may be more chronic. Our results are consistent with prior studies that reported more pronounced cortical thinning and volume loss in treatment-resistant patients compared to non-treatment-resistant patients^64,65^. In treatment-resistant patients, the relationship remained significant even after controlling for illness severity and CPZ antipsychotic drug dose equivalents. The ROI analyses also identified an association between impaired general illness awareness (i.e., VAGUS general illness awareness domain) and cortical thinning in the left supramarginal gyrus. We found no associations between impaired insight and subcortical volumes.

A previous study by our group that examined the structural and functional correlates of insight found a direct link between cortical thinning and brain activity. In this study we found that higher brain activity in the left posterior parietal area during a functional MRI insight task in relation to insight impairment was associated with cortical thinning in the same region^60^. The results of other studies that have focused on examining the relationship between impaired insight and cortical thickness have been inconsistent^14^^,17–19,66^. The inconsistencies in the literature may be due to a number of factors, including limited sample size and different methods of measuring insight into illness^14,17–19^. To address some of these limitations, a study by Béland et al. included a large number of patients with schizophrenia (*N* = 110) to examine the association between clinical insight and cortical thickness. In their study, insight was determined by calculating two statistically derived factors from four different measures of insight. The two factors that emerged were: (i) awareness of illness and need for treatment, and (ii) awareness of symptoms and consequences of the disorder. The authors examined 8 brain regions previously implicated in impaired insight, including the inferior parietal area, but did not find any relationship between cortical thickness and the two insight factors^19^. There are several differences between Béland et al. and the present study that may explain the differences in the results. First, approximately half of the participants in the current study were on clozapine and considered to have treatment-resistant schizophrenia. Therefore, it is likely that our sample included more chronically ill patients with persistent insight deficits in comparison with the sample in the Béland et al. (2019) study. A separate investigation that included patients with first-episode psychosis, chronic, and treatment-resistant schizophrenia revealed extensive cortical thinning in brain regions with higher structural connectivity. The strength of structural connectivity between regions with cortical thinning increased with illness severity and was the highest in treatment-resistant schizophrenia^67^. Plausibly, cortical thinning in brain regions implicated in insight impairment is more apparent in treatment-resistant schizophrenia due to prolonged aberrant connectivity between these interconnected brain regions^59,68^. Second, we used a single measure of insight into psychosis rather than statistically derived factors. However, the VAGUS scale measures the same core domains of illness awareness, specifically general illness awareness, symptom attribution, awareness of the need for treatment, and awareness of negative consequences of the disorder^23^. Although there is evidence to suggest the neurobiological basis of insight is specific to insight types and its dimensions^69^, the relationship between insight impairment and cortical thinning in our study was mainly observed with VAGUS average scores. Only the VAGUS general illness awareness subscore was found to be associated with cortical thinnning in the left supramarginal gyrus in our ROI analyses.

Analogous to anosognosia, a neurological phenomenon characterised by deficits in illness awareness resulting from parietal and/or frontal lesions, impaired insight in patients with schizophrenia has been proposed to arise from deficits in these same brain regions^12,13,70–74^. Studies in patients with first-episode psychosis showed reduced right dorsolateral prefrontal cortex volume in patients with impaired insight^71,73^. Similarly, a longitudinal study found that volume deficits in frontal and parietal regions in patients with first-episode psychosis predicted insight impairments two years after the onset of the first-episode^75^. Consistent with these results, our study found cortical deficits in frontal and parietal regions, specifically the left inferior and middle frontal gyrus and the supramarginal gyrus, in patients with impaired insight. Cortical thinning was also observed in the left temporal pole. The left inferior frontal region is frequently implicated in self-referential processing where several functional neuroimaging studies in healthy controls and patients with schizophrenia have shown increased activity in this region during self-reflection tasks^76,77^. Moreover, a previous functional neuroimaging study by our group in patients with schizophrenia found an association between greater self-certainty and reduced resting-state functional connectivity with the left inferior frontal cortex in the dorsal attention network, suggesting a deficit in this region may reflect a lack of mental flexibility in patients with impaired insight^59^. The temporal pole, a component of the paralimbic circuit along with the orbitofrontal cortex and the insular cortex, is engaged in various cognitive functions including language, multisensory integration, and social and emotional behaviours, all of which have been found to be impaired in patients with schizophrenia^78,79^. Its direct link with impaired insight requires further exploration.

A number of investigations suggest that impaired insight in patients with schizophrenia likely arises from disrupted brain networks rather than solely from deficits in specific brain regions^18,68,80,81^. Resting-state functional connectivity studies have shown aberrant connections within the default mode network and the self-reflection network in patients with impaired insight^59,82^. A diffusion tensor imaging study by our group also showed reduced posterior corpus callosal tract integrity in patients with impaired insight compared to those with intact insight and healthy controls, possibly reflecting disrupted communication between key brain regions associated with impaired insight^68^.

Although our study did not find a statistically significant association between impaired insight and thalamic volume, we suspect our study may have been underpowered to detect this relationship. We theorise that cortical abnormalities in frontoparietal regions in patients with impaired insight may reflect deficits in the indirect frontoparietal pathway that connects the frontal and posterior parietal regions via the basal ganglia and the thalamus^83–85^. Further studies are needed to examine the links among cortical and subcortical structural and functional deficits in patients with impaired insight in schizophrenia.

The present study has several limitations. First, this study was conducted retrospectively. As a result, measures that would have helped further validate our results, such as using a clinician-rated measure of insight, were not collected. The lack of a clinician-rated measure is somewhat mitigated by the strong correlation between the VAGUS-SR and the clinician-rated version and other established clinician-rated measures of insight into illness in schizophrenia^23^. Arguably, self-report measures obviate clinician factors, such as bias, that may negatively influence the accurate assessment of insight into illness in schizophrenia^86,87^. Second, we did not compare treatment-resistant and non-treatment resistant participants as our variable of interest was insight into illness and to do so adequately would require a sophisticated matching analysis based on degree of insight and other relevant variables. Third, due to the cross-sectional design of the study, we were unable to account for the effects of prior antipsychotic drug use (e.g., specific drug and duration of use) on cortical thickness and subcortical volumes. As all of the treatment-resistant patients were on clozapine, it is possible that clozapine contributed to cortical thinning in these patients^88,89^. That being said, there is more evidence to support the theory that illness chronicity or resistance contributes to cortical thinning in schizophrenia^20–23^. Last, the sample size in the non-treatment-resistant group was relatively small. Therefore, it is possible that we did not have enough statistical power to detect significant associations in this group. Relatedly, there were likely patients in the non-treatment-resistant group who are non-responsive to their current treatment but did not meet our criteria for treatment resistance.

## Conclusions

Overall, our findings suggest that cortical thinning in the left frontal, temporal pole, and posterior parietal regions is associated with insight impairment in patients with a schizophrenia spectrum disorder, and more so in patients with treatment resistance. Future longitudinal studies with well-characterised samples representative of the full spectrum of insight and illness severity may be needed to better understand the neuroanatomical correlates of insight into illness in schizophrenia.

## Supplementary information

Supplemental Material
