# Supplementary material for: Cortical thinning in relation to impaired insight into illness in patients with treatment resistant schizophrenia
Source: Schizophrenia (Heidelb). 2023 Apr 29;9(1):27. doi: 10.1038/s41537-023-00347-y (PMC10148890; doi:10.1038/s41537-023-00347-y)
Supplement: Supplementary file 1 — Supplemental Material [file 41537_2023_347_MOESM1_ESM.docx]

**Supplemental Material 1.** Correlations between VAGUS scores and participant demographic and clinical characteristics

|  | VAGUS  average score | VAGUS  general illness awareness | VAGUS  symptom attribution | VAGUS  awareness of need for treatment | VAGUS  awareness of negative consequences |
| --- | --- | --- | --- | --- | --- |
| Age | r(94)=0.16, p=0.177 | r(94)=0.22, p=0.038 | r(90)=-0.11, p=0.308 | r(93)=0.25, p=0.015 | r(94)=0.14, p=0.177 |
| Age of onset | r(94)=0.00, p=0.978 | r(94)=-0.11, p=0.281 | r(90)=0.11, p=0.305 | r(93)=0.00, p=0.983 | r(94)=0.04, p=0.726 |
| Illness duration | r(94)=0.17, p=0.112 | r(94)=0.26, p=0.013 | r(90)=-0.17, p=0.113 | r(93)=0.24, p=0.020 | r(94)=0.16, p=0.121 |
| Education (years) | r(93)=0.02, p=0.879 | r(93)=-0.04, p=0.698 | r(89)=0.08, p=0.436 | r(92)=-0.10, p=0.366 | r(93)=0.07, p=0.493 |
| WTAR | r(88)=0.02, p=0.853 | r(88)=0.10, p=0.333 | r(84)=-0.04, p=0.727 | r(87)=-0.10, p=0.366 | r(88)=0.05, p=0.629 |
| PANSS total modified $\dagger$ | r(94)=-0.21, p=0.043 | r(94)=0.00, p=0.999 | r(90)=-0.43, p<0.001 | r(93)=-0.14, p=0.185 | r(94)=0.026, p=0.805 |
| PANSS positive | r(94)=-0.19, p=0.070 | r(94)=0.04, p=0.726 | r(90)=-0.45, p<0.001 | r(93)=-0.13, p=0.207 | r(94)=0.06, p=0.601 |
| PANSS negative | r(94)=-0.28, p=0.007 | r(94)=-0.14, p=0.181 | r(90)=-0.25, p=0.024 | r(93)=-0.13, p=0.232 | r(94)=-0.19, p=0.067 |
| PANSS general$\dagger$ | r(94)=-0.12, p=0.238 | r(94)=0.08, p=0.466 | r(90)=-0.35, p=0.001 | r(93)=-0.12, p=0.272 | r(94)=0.07, p=0.505 |
| CPZ dose (mg/day) | r(94)=-0.15, p=0.142 | r(94)=-0.16, p=0.136 | r(90)=-0.15, p=0.150 | r(93)=-0.06, p=0.570 | r(94)=-0.05, p=0.650 |

WTAR, Wechsler Test of Adult Reading; PANSS, Positive and Negative Syndrome Scale; CPZ, chlorpromazine; $\dagger$PANSS score minus item G12

**Supplemental Material 2.** List of antipsychotic medications

Clozapine (n=56)

Olanzapine (n=12)

Aripiprazole (n=5)

Flupentixol (n=4)

Fluphenazine decanoate (n=1)

Haloperidol (n=1)

Loxapine (n=1)

Lurasidone (n=1)

Paliperidone (n=6)

Perphenazine (n=1)

Quetiapine (n=2)

Risperidone (n=3)

Ziprasidone (n=1)
